# The Genomic Landscape of Metallic Colour Variation in Ground Beetles

**DOI:** 10.1111/mec.70591

**Published:** 2026-10-16

**Authors:** Yi‐Ming Weng, David H. Kavanaugh, Bryan Rubio‐Perez, Jad Salman, Mikhail A. Kats, Sean D. Schoville

**Affiliations:** ^1^ Department of Entomology University of Wisconsin‐Madison Madison Wisconsin USA; ^2^ Okinawa Institute of Science and Technology Graduate University Okinawa Japan; ^3^ Department of Entomology California Academy of Sciences San Francisco California USA; ^4^ Department of Electrical and Computer Engineering University of Wisconsin‐Madison Madison Wisconsin USA; ^5^ Department of Physics University of Wisconsin‐Madison Madison Wisconsin USA; ^6^ Department of Material Science and Engineering University of Wisconsin‐Madison Madison Wisconsin USA

**Keywords:** functional ecology, insects, metallic colour, *Nebria*, phenotypic evolution, wild‐GWAS

## Abstract

The metallic colour variation of beetles is a spectacular feature that has inspired diverse human cultures. However, little is known about the genetic basis of this trait or its ecological importance. In this study, we characterize the geographical distribution, optical mechanism, genetic basis and ecological and evolutionary importance of metallic colour variation in the *Nebria ingens* complex, an alpine ground beetle in the Sierra Nevada, California. We find that elytral colour varies continuously across two allopatric species (from black *N. ingens* to green *N. riversi*), with hybrid populations showing intermediate coloration, and we demonstrate that the metallic colour is generated from multilayer reflectors in the epicuticle of the elytra. By applying association mapping in natural populations (wild‐GWAS) using high‐density genotype variants, we identify five promising candidate genes covarying with metallic variation, with known roles in cuticle formation and pigmentation pathways. This finding, together with a significant correlation between colour variation and water availability, suggests that metallic variation evolves as a local adaptation to environmental variation in the *N. ingens* complex.

## 1 Introduction

The impression created by bright metallic coloration in beetle wings is so stunning that diverse human cultures have used them in art and jewellery (‘beetlewing art’) for thousands of years (Ratcliffe 2006; Rivers et al. 2003). This remarkable phenotypic trait is all the more impressive when its tremendous variation among beetles (order Coleoptera), a biodiverse group including more than 500,000 species, is recognized to have repeatedly evolved (Seago et al. 2009). The ecological significance of metallic coloration is generally thought to be associated with inter‐ and intraspecific interactions, such as species recognition, mimicry and aposematism (Muñoz‐Ramírez et al. 2016; Seago et al. 2009). However, relatively little attention has been given to the potential relationship between metallic coloration in beetles and adaptation to local environmental conditions. This is particularly intriguing given that colour variation, including both intraspecific variation and reversible colour changes within individuals, has been shown to be associated with water availability in multiple beetle species across diverse lineages (Rassart et al. 2009; Vigneron et al. 2007; F. Liu et al. 2009; Hull‐Sanders et al. 2003; Sun et al. 2021). Other the other hand, the optical mechanism of metallic colour in insects is well known, generated by the internal structure of the exoskeleton (Andersen 2009). In Coleoptera, the most prevalent internal structure is a multilayered reflector in the epicuticle or exocuticle, where the layers with alternating refractive index reflect light in a specific range of wavelengths (Barry et al. 2020; Seago et al. 2009). For species exhibiting multilayered reflectors, variations may involve differences in saturation (chroma), hue, or both. The thickness of the alternating layers primarily governs the hue, with thicker layers producing longer peak wavelengths and thinner layers resulting in shorter ones (Kurachi et al. 2002). Meanwhile, saturation can be influenced by the number of layers or the overall tanning level of the cuticle. As a result of the discovery of the optical mechanism of metallic colours in insects, these microstructures have become an important resource for bioinspired materials (Biró et al. 2009; Biró and Vigneron 2011; Colusso et al. 2017).

Although metallic coloration is known to be polymorphic in numerous beetle species (Kozlov et al. 2022; Strickland et al. 2019), little is known about the genetic basis of this phenotypic trait (Endler and Mappes 2017). Compared to beetles, the genetic basis of wing coloration in butterflies has been more extensively studied and could provide hypotheses for testing the roles of similar genes in regulating multilayer reflectors in beetles. For example, transcription factors such as *optix*, *Antennapedia*, *aristaless1*, *bric‐a‐brac* (*bab*) and *spalt* are involved in various pigmentation pathways and in regulating the photonic microstructures on butterfly scales (Ficarrotta et al. 2022; Livraghi et al. 2025; Mazo‐Vargas et al. 2022; Prakash et al. 2022; Reed et al. 2020; Thayer et al. 2020; Zhang, Mazo‐Vargas, and Reed 2017; Zhang, Martin, et al. 2017). These transcription factors may also regulate the synthesis, transport, or deposition of melanized layers in the beetle cuticle. On the other hand, genes involved in the tyrosine‐mediated tanning pathway such as *ebony*, *tan*, *arylalkylamine N‐acetyltransferase* (*aaNAT*), *laccase2*, *yellow*, *pale* (*tyrosine hydroxylase*), *Dopa decarboxylase* (*DDC*), along with potential transcription factors such as *pannier* (*pnr*) and *POU domain motif 3* (*pdm3*), are responsible for the production and distribution of melanin pigments and could therefore contribute to patterning the multilayer reflector (Arakane et al. 2016; Niimi and Ando 2021).

Since studies of diverse beetle species have shown that metallic colour variation is typically additively determined by a small number of genetic loci (Favila et al. 2000; Fujiyama and Arimoto 1988; Gotoh and Lavine 2014; Mossakowski and Paarmann 2014; Vasconcellos‐Neto 1988), and only infrequently dependent on genotype‐environment interactions (Davis et al. 2008), it should be possible to identify the genes underlying the metallic colour variation by association approaches (Wellenreuther and Hansson 2016).

In this study, we aim to investigate the genetic basis of metallic colour from the multilayer reflector in the cuticle of alpine ground beetles, the *Nebria ingens* species complex, by using whole genome sequence data and association mapping in natural populations (wild‐GWAS). The *N. ingens* species complex is endemic to the Sierra Nevada in California, where it is found in the high elevation nival zone that is covered by snow throughout most of the year. Two species with divergent colour in their elytra (appearing metallic green or black to human perception), *N. ingens* Horn and *N. riversi* Van Dyke, are geographically isolated, but form a broad admixture zone of intermediately coloured individuals (Schoville et al. 2012; Weng, Kavanaugh, and Schoville 2021; Weng, Francoeur, et al. 2021) (Figure 1). In addition, microhabitat conditions gradually vary latitudinally, from the snow‐covered habitats of northern *N. riversi* to the drier microhabitats occupied by southern *N. ingens* (Weng et al. 2024). The morphologically variable and genetically admixed populations inhabiting these gradually changing microhabitats make this species complex a great study system to apply wild‐GWAS approaches (Buerkle and Lexer 2008; Lindtke et al. 2013). Replicate admixed populations disrupt linkage disequilibrium across the genome, increasing power to isolate causative loci underlying the divergent phenotypes of the two species. Furthermore, the nearly continuous colour variation improves the statistical power of association analyses. Leveraging this system, we identify candidate for the genetic basis of metallic colour in beetles which could be tested in the future expression regulation or gene editing approaches.

**FIGURE 1:**
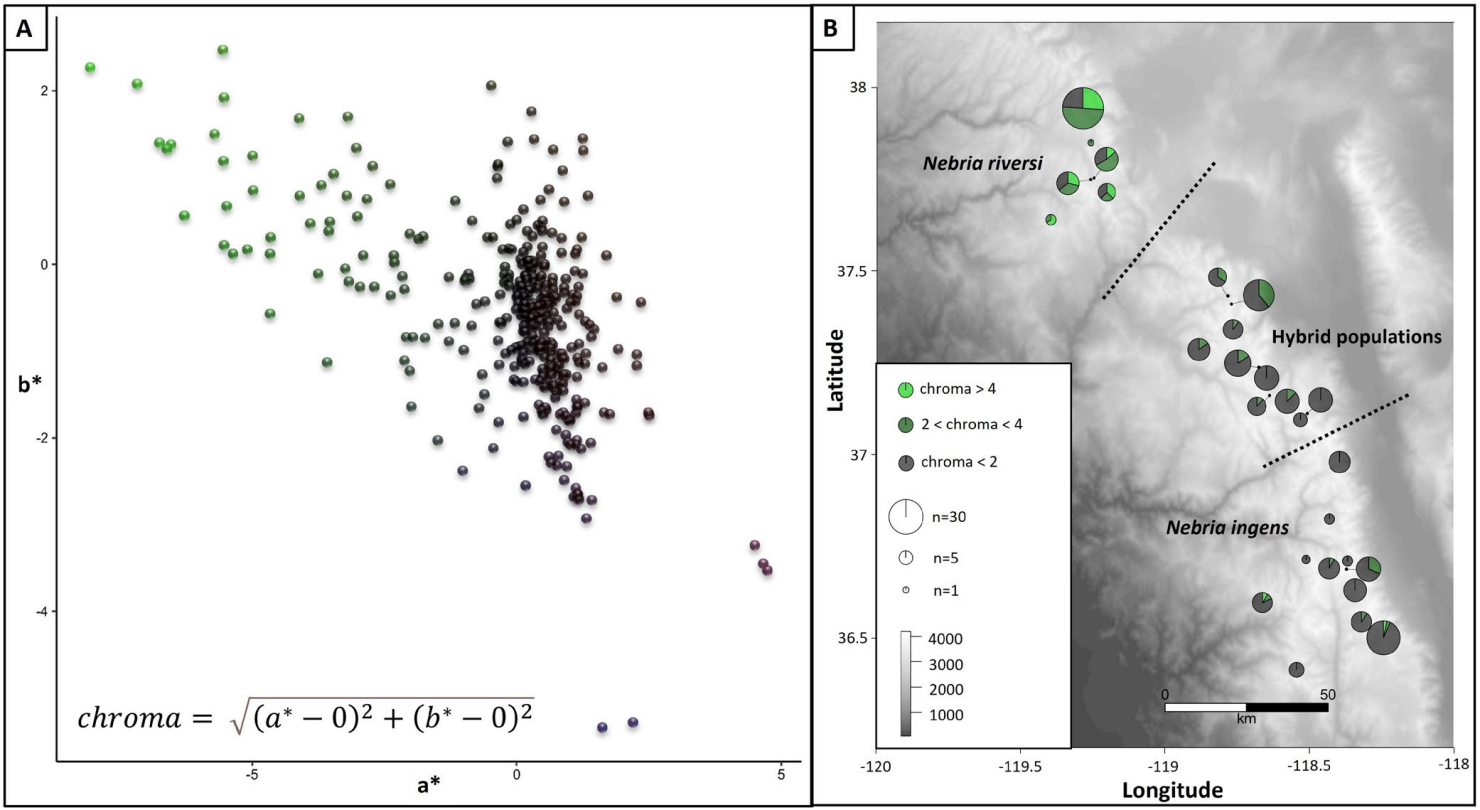
Colour variation of the elytra in the *Nebria ingens* species complex. (A) Quantification of colour using the a^⁎^ (green‐red axis) and b^⁎^ (blue‐yellow axis) colour space, where chroma is defined as the distance of each point from the achromatic centre (0,0). In the scatterplot (A), each dot represents an individual beetle and the colour of each dot represents the reconstructed beetle coloration based on the combination of the green–red and blue–yellow colour gradients. (B) Map showing the geographic distribution of colour variation across sampling sites: The northernmost six populations correspond to *N. riversi*, the southernmost eleven populations to *N. ingens* and the intermediate populations represent admixed groups.

## 2 Materials and Methods

### 2.1 Animal, Genotyping and Colour Variation

Adult beetles of the *N. ingens* complex (*n* = 404) were collected from 27 populations, encompassing the entire distribution range of the species complex (Figure 1; Table S1). The genome‐wide variants were identified from the whole‐genome resequencing data of 374 beetles in our previous study using the GATK variant calling pipeline, where WGS reads were mapped to a reference genome assembly of *N. riversi* resulting in 7,380,667 raw variants (Weng, Francoeur, et al. 2021; Weng et al. 2024). In this study we applied hard‐filters to the original cohort VCF file to generated the high‐quality SNP and indel (insertion and deletion) variants (details see Supplementary methods), where indels were masked with A/G for reference and alternative allele for downstream analyses (Van der Auwera and O'Connor 2020; Weng et al. 2024). After removing samples lacking colour phenotypic data and loci with minor allele frequency less than 0.05 using vcftools v0.1.16 (Danecek et al. 2011), we performed GWAS using a genotype matrix with 355 beetles and 526,717 SNP + indel variants, with kinship coefficients calculated using the R package SNPRelate to ensure the beetle samples are unrelated (Table S2). To quantify optical variation, we measured the reflectance spectra from the elytral surface of 368 beetles using a hyperspectral camera (Specim IQ) (Table S3). The average spectrum was measured from each pixel in the brightest elytral region (8–600 pixels, median = 190 pixels) to represent the individual reflectance spectrum. The mean reflectance spectrum was then used to calculate the CIELAB colour for each elytron (Robertson 1977) using the CIE standard illuminant D65 with a custom MATLAB script. Chroma was calculated as the distance from the neutral grey point (0,0) in the a*b* plane to represent the colour saturation of the elytra (Table S4).

### 2.2 Cuticular Structure of the Metallic Hue

The elytra of three beetles, representing *N. ingens* , *N. riversi*, and a genetically admixed hybrid lineage, were used to examine the cuticular microstructure with transmission electron microscopy (TEM) to investigate whether differences in the multilayer structure of the epicuticle could account for the observed differences in their colour variation and reflectance spectra. We used a FEI Tecnai T12 microscope in the School of Medicine and Public Health at UW‐Madison. The samples were stored in −20°C with 95% ethanol before use. Images showing clearly resolved structure were selected to measure the thickness of the epicuticle. We took 26–30 vertical transects to repeat measurements of thickness for each layer composing the epicuticle using ImageJ, where the epicuticle was distinguished from the underlying exocuticle based on differences in layer thickness and contrast in the TEM images (Abràmoff et al. 2004) (Table S5). Counts and thickness of layers were used to predict the reflectance spectra (i.e., calculated reflectance spectra) of each specimen using a multilayer reflectance calculator provided by Filmetrics Corporation, with the refractive indices of electron dense and electron lucent being 1.7 and 1.4, respectively (Figure 2). These indices are consistent across different beetle taxa (Bernard and Miller 1968; Kurachi et al. 2002). The calculated reflectance spectrum was subsequently used to compare with the observed spectra (the reflectance spectra directly measured from the samples) from the same individual beetle, except for the green elytrum for which we compared the calculated spectrum to other bright green beetles from the same population, to verify the source of the metallic colour (Figure 2) (Table S6).

**FIGURE 2:**
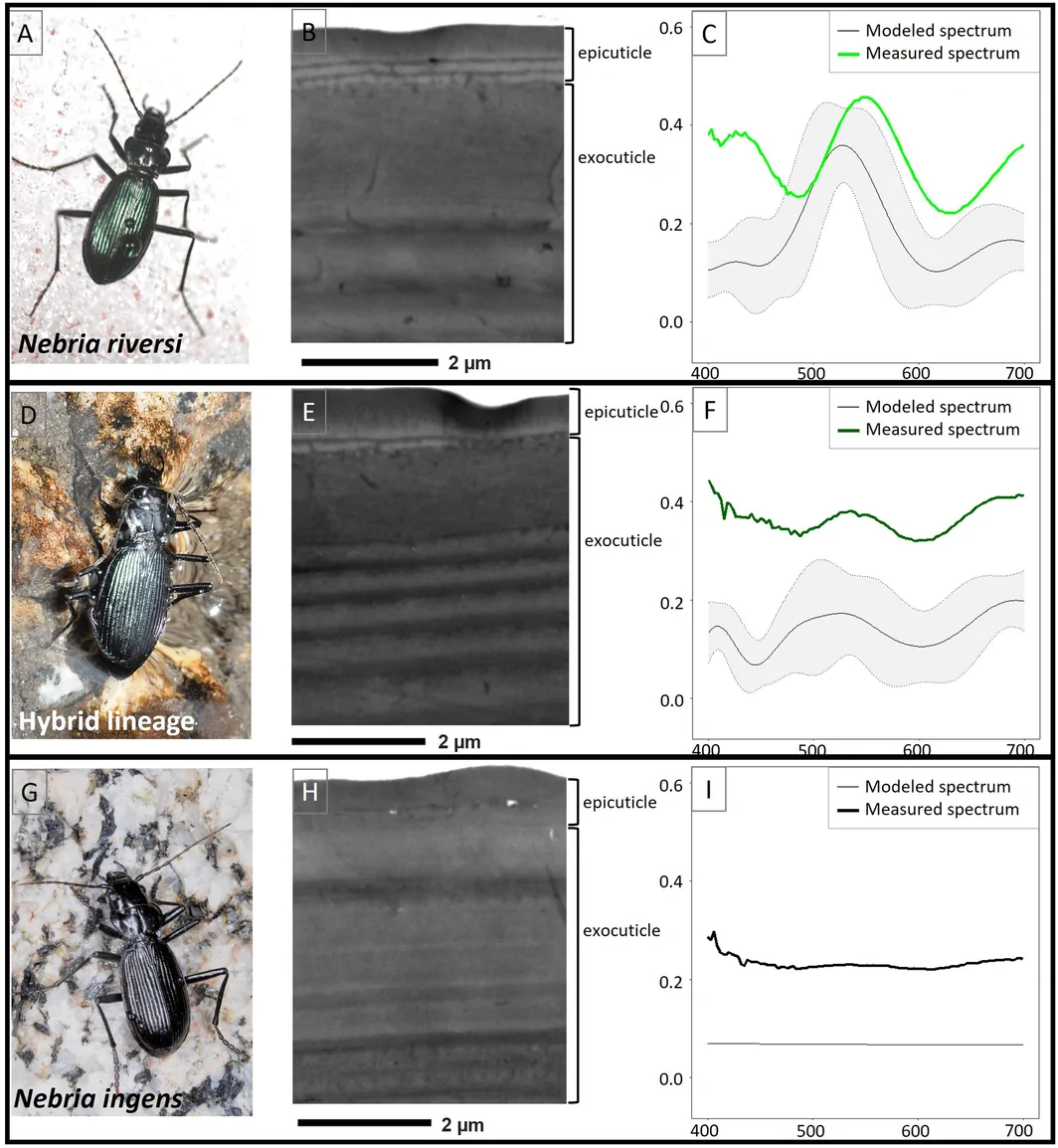
Comparisons of modelled spectra calculated from measurements of the multilayer reflectors in the epicuticle of individuals with observed reflectance spectra in different elytral colours (A–C: *Nebria riversi* (green), D–F: Hybrid lineage (dark green), G–I: *Nebria ingens* (black)). The TEM images (B, E, H) demonstrate the pattern of multilayer reflectors in the epicuticle. In each spectrum plot (C, F, J), the thin lines represent the means of modelled spectra from different observation points (see Supplemental methods) and the grey area shows the 95% confidence interval from measurements of different vertical transects, and the measured spectra are denoted with thicker lines.

### 2.3 Genome‐Wide Association Mapping

To identify SNP and indel markers associated with the colour phenotype, we performed GWAS using latent factor mixed models (LFMM) and partial redundancy analysis (pRDA) implemented using R package lfmm v1.1 and vegan v2.7–1, respectively (Caye et al. 2019; Oksanen et al. 2025). In LFMM analysis, four latent factors were specified to account for population structure, which includes a pattern of isolation by distance across seven ancestral population clusters determined by the population clustering analyses with genome‐wide SNP data in our previous study (Kavanaugh et al. 2021). The regularized least squares estimate of causal variants were obtained using both ridge and lasso regression. To reduce the false‐discovery rate (FDR), we first calibrated the *p* values with genomic inflation factors (GIF), then adjusted the calibrated *p* values using the Benjamini‐Hochberg multiple‐testing procedure. Variants with the adjusted *p* value less than 0.01 in ridge or lasso regression were used to check the constancy between the two regression methods and were considered strong candidates. In pRDA analysis, genotype data was first transformed into plink format (.raw) using vcftools v0.1.16 (Danecek et al. 2011). To adjust the linear effects of chroma on genotype while accounting for the variation from the population structure, the first four PC axes calculated with SNPRelate v1.42.0 were included as conditioning variables, as the first four PCs were shown to result in clear population clusters in previous work (Zheng et al. 2012; Weng et al. 2024). Variants falling outside of four standard deviations of first RDA axis loading are considered candidate variants for chroma. The intersection of candidate variants from the two analyses were used to find the intersecting genes from the published genome using bedtools v2.28 (Quinlan 2014; Weng, Francoeur, et al. 2021). Gene intersection was defined by variants falling into the genetic region and their 2 kilobase upstream (5′) flanking region, as regulatory elements could be located thousands of base pairs from the 5′ flanking region. We focused on the 5′ flanking regions, as the variants affecting promoter‐proximal cis‐regulatory elements could alter the expression of genes encoding enzymes involved in the tyrosine‐mediated melanization pathway. Finally, we identified the annotated function of the candidate genes based on their best blast hit against the NCBI nr database and InterPro annotations generated using InterProScan v5.76–107.0 (Blum et al. 2025; Camacho et al. 2009; Jones et al. 2014), and compared candidate genes with our previously published genes with signal of selective sweeps from OmegaPlus to identify overlapping genes potentially involved in both colour variation and evolutionary adaptation (Alachiotis et al. 2012; Weng et al. 2024).

### 2.4 Testing for Environmental Selection on Beetles' Colour Variation and Water Retention

Because colour variation is a key diagnostic character separating two species in the *N. ingens* complex, and microhabitat conditions vary along a latitudinal gradient in terms of summer snowpack, colour variation may be associated with adaptation to local environments. To test the hypothesis that microhabitat conditions are associated with water‐retention ability through colour variation, we conducted two regression analyses: one testing the relationship between beetle colour variation and the snow water equivalent (SWE) of sampling sites, and the other testing the relationship between beetle water‐loss rate and colour. Habitat SWE data (represented by measurements of snow water equivalent in September) were extracted from published Sierra Nevada snow pack analysis results using the R package raster v3.6–3, with further methodological details provided in our previous study (Margulis et al. 2016; Weng et al. 2024). For water loss rate, 87 beetles from five sample sites were placed individually in a tube in a sealed container, which contained lithium chloride (LiCl) as a desiccant to maintain a relative humidity between 20% and 30%. The temperature was controlled at 5°C during the experiment, which is similar to the natural foraging temperature in the field. The water loss rate was calculated by weighing beetles before and after 24 h of desiccation. For regression analyses, we first tested the effect of SWE on beetle coloration (chroma) using a linear mixed‐effects model (LMM) with the lme4 v1.1.37 package, where sample site was included as a random effect and square root transformation was applied to chroma to address the normality of the residual (Bates et al. 2015). The fixed‐effect *p*‐value was calculated using lmerTest v3.2.1, where the degree of freedoms were estimated using Satterthwaite's approximation (Kuznetsova et al. 2017). For the test of whether chroma predicted water loss rates of the beetle, we used a least squares linear regression, as the samples with water loss rate data were collected from only five sites.

## 3 Results

### 3.1 Continuous Variation and the Mechanism Controlling Metallic Colour

We examined 368 beetles from 27 populations across the distribution of the species complex. In general, the reflectance spectra of green beetle elytra have a peak of 500 to 600 nm wavelength, while beetles with black elytra have a lower reflectance amplitude and loss of the characteristic reflectance peak resulting in the green colour (Figure 2). By converting the reflectance spectra to the CIELAB colour space (L*a*b*) using the CIE standard illuminant D65, we found that many, but not all beetles identified as *N. riversi* reflect a green hue, while most beetles identified as *N. ingens* or as hybrids reflect a dark green to black hue (Figure 1). The colour variation is continuously distributed and thus likely to be a quantitative trait encoded by multiple genes.

From the elytral cross section, TEM images of green, dark green and black elytra demonstrate variation in the inner epicuticle (cuticulin layer), which is most pronounced in the green elytra (Figure 2). There are 1–3 electron dense layers 50–100 nm thick (darker layers in the images due to reduced permeability for the electron beam), alternating with similar‐width electron lucent layers, serving as a multilayered reflector (Figure S1). In the dark green elytra, only one electron dense layer is found, and no clear multilayer reflector was observed in the black elytra throughout the examined regions. Calculated spectra based on the thickness of the multilayer reflectors generally match the visual colour and observed spectra, with more alternating layers producing a stronger amplitude of light at a peak wavelength between 500 and 600 nm, roughly corresponding to the colour green (Figure 2).

### 3.2 Colour Associated Genes and Their Roles in Colour Determination

Among the 526,717 scanned variants, 892 (0.17%) passed the adjusted *p*‐value threshold at 0.01 in ridge or lasso regression models with high consistency between the two regressions (R‐squared = 0.95; Figure S2) and 573 (0.11%) fall outside of the 4th standard deviation of the first RDA axis loading distribution (Table S7). The intersection of the two approaches contains 195 (0.037%) variants which intersect 127 genes including 65 genes with functional annotations (Table S8) and five with known functions in different pathways of insect cuticle formation and pigmentation (Figure 3). These include *yellow‐e* and *pdm3* in the tyrosine‐mediated tanning pathway, *aristaless* in the ommochrome pathway, *chico* (encoding insulin receptor substrate (IRS) protein) in the Insulin/IGF signalling pathways through PI3K/Akt, and a gene encoding Pyrokinin‐1 receptors‐like protein (*PK1*‐like), a type of G‐protein coupled receptor in the pyrokinin signalling pathway (Matsumoto et al. 1990; Shakhmantsir et al. 2014). All five genes have intersecting variants within intragenic regions, and the variants in *pdm3* and *chico* are in the coding region. Finally, the candidate genes *chico*, *PK1*‐like, and small G protein signalling modulator 3 (*GPSM3*) homologue were previously identified in genome‐wide scans for selective sweeps.

**FIGURE 3:**
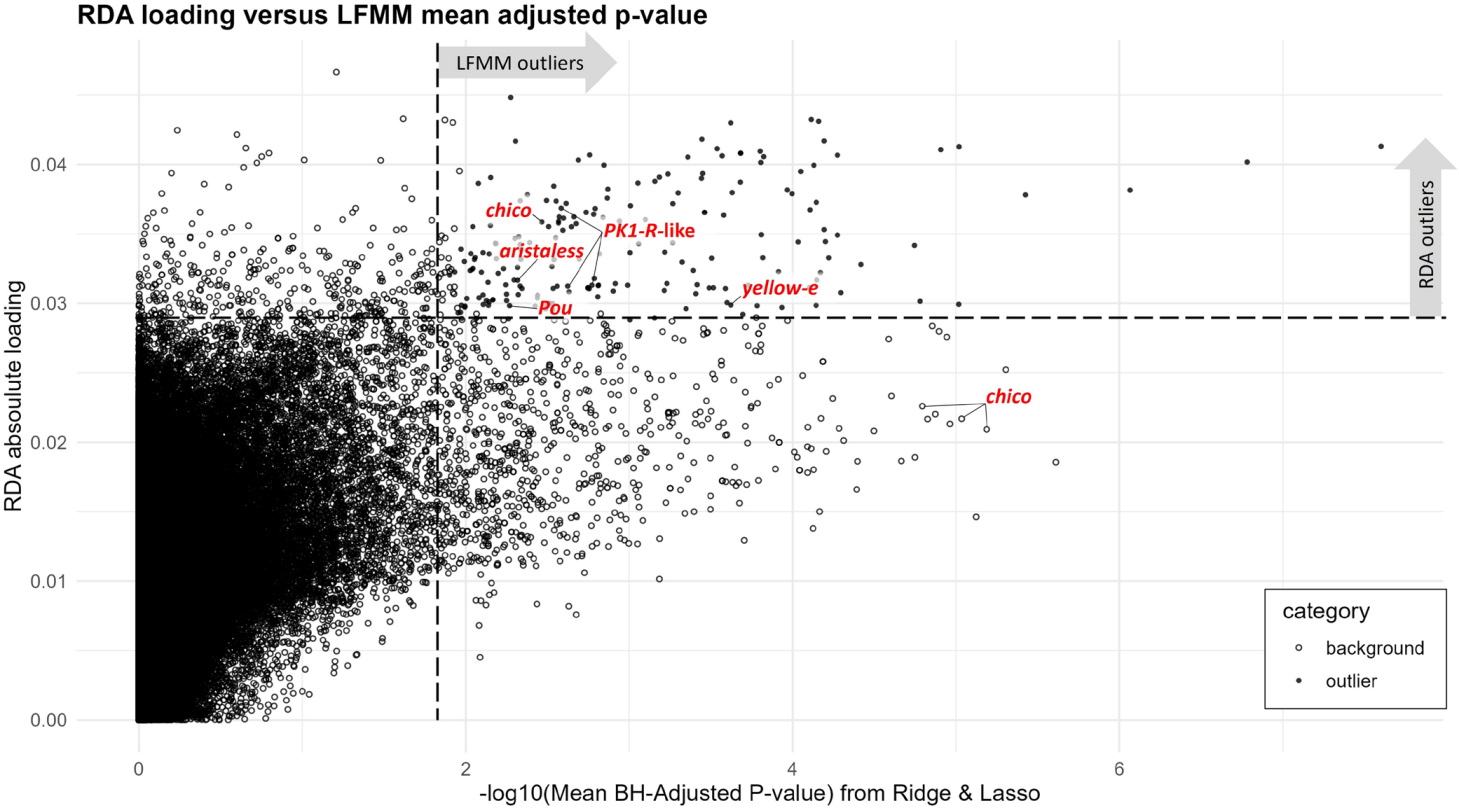
The scatter plot with mean BH‐adjusted *p*‐value of LFMM (x axis) and RDA absolute loading (y axis). Each dot represents a variant where the close dots are the outlier (candidate) variants defined by LFMM BH‐adjusted *p*‐value of ridge or lasso less than 0.01, and RDA loading falling outside of the 4th standard deviation.

### 3.3 Association Between Colour Variation, Water Loss Rate and SWE

To test the physiological and ecological function of metallic colour, we performed regression analyses using chroma, water loss rate and seasonal water availability in the nival zone (Figure 4). The LMM shows that beetle coloration (chroma) was positively associated with the snow water equivalent (SWE) of sampling sites (t = 5.58; *p* = 2.51 × 10^−6^), indicating that beetles from sites with higher SWE tended to exhibit higher chroma values. Water‐loss rate also showed a positive relationship with beetle coloration, but this relationship was not statistically significant at the conventional threshold (*p* = 0.0516; R^2^ = 0.068). Thus, water‐loss rate tended to increase with chroma (*p* = 0.0516; R^2^ = 0.068) but chroma explained only 6.8% of the variation in water‐loss rate.

**FIGURE 4:**
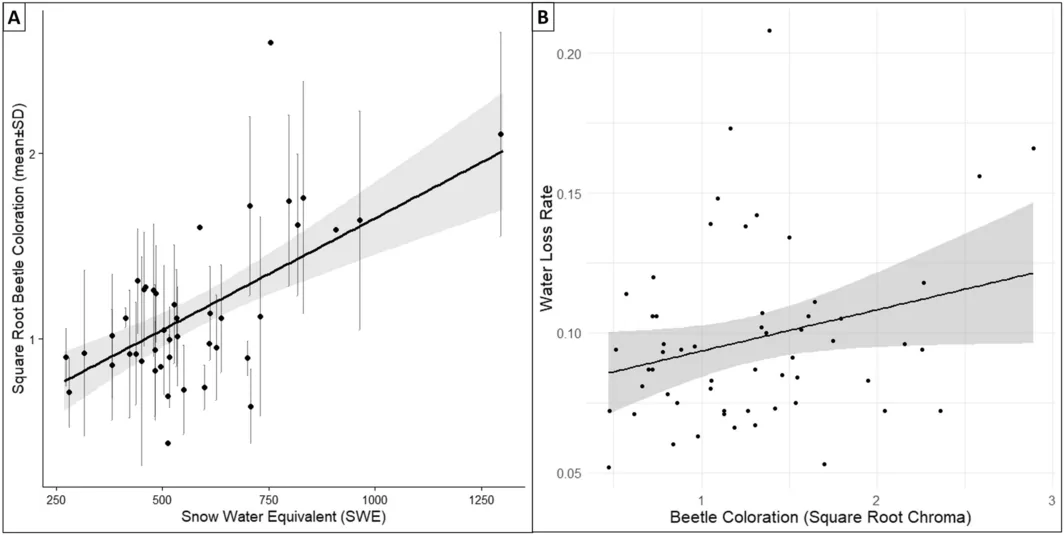
(A) Relationship between sample site snow water equivalent (SWE) and square‐rooted beetle coloration (chroma) and (B) square‐rooted chroma and water loss rate. In (A), points represent site‐level means aggregated from individual measurements with one standard deviation as error bars. The trend line indicates the site‐level fixed effect and 95% confidence intervals generated directly from the linear mixed‐effects model (LMM) using the individual‐level data. In (B), points indicate individual beetle observations and the trend line represents the best‐fit linear regression and corresponding 95% confidence intervals derived from the ordinary least squares linear model.

## 4 Discussion

Despite a long‐standing interest in the metallic colour of insects, distinct knowledge gaps in our understanding of colour variation remain. Beyond the lack of knowledge for the most biodiverse animal group, the beetles, gaps include the nebulous genetic basis of metallic colour variation. By applying wild‐GWAS to an alpine ground beetle species complex with admixed populations, we found promising candidate genes that are statistically associated with the metallic variation generated from multilayer reflectors in the epicuticle and functionally involved in cuticular structure and melanization. These include genes from several known pathways involved in butterfly colour patterns.

### 4.1 Genetic Basis of the Metallic Colour in the *N. ingens* Complex

Colour variation can be categorized by optical mechanisms which vary across insect lineages. For instance, in Coleoptera, iridescent colour generated by three‐dimensional photonic structures has been found in weevils and longhorn beetles, whereas multilayer reflectors are widespread in distantly related lineages of jewel beetles (Buprestidae), ground beetles (Carabidae), leaf beetles (Chrysomelidae) and scarab beetles (Scarabaeidae) (Seago et al. 2009, 2019). Although structural layering may be common to the ancestors of beetles, structural coloration has been repeatedly gained, lost and modified (Seago et al. 2019). Yet very little is known about how genetically complex this phenotypic variation is across beetles.

In this study, we found that a multilayer reflector is responsible for the metallic colour variation of the *N. ingens* complex. This is directly supported by the presence of the layers with alternating refractive index in the epicuticle, with their estimated reflectance spectra matching the elytral colours and the observed spectra (Figure 2). Such resemblance of the calculated and observed reflectance spectra has been used to determine the optical mechanism of metallic colour in many other insects (Bermúdez‐Ureña et al. 2020; Kurachi et al. 2002; J. Lee et al. 2020; Luna et al. 2013; Noyes et al. 2007; Parker et al. 1998; Pasteels et al. 2016). In the *N. ingens* complex, the amplitude of the peak between 500 and 600 nm in the reflectance spectrum is directly correlated with the number of alternating layers in the epicuticle, representing a form of saturation variation characterized by changes in amplitude at specific wavelengths caused by differing numbers of layers with consistent thickness. It is structurally distinct from phenotypes where the reflectance peak shifts across wavelengths due to differences in layer thickness and therefore should be regarded as a separate category of colour variation. In nature, this type of saturation variation seen in the *N. ingens* complex appears to be less common but has been reported in different beetle families (Gotoh and Lavine 2014; Mossakowski and Paarmann 2014; Stanbrook et al. 2021).

Knowing that the metallic colour variation in the *N. ingens* complex is regulated by multilayer reflectors, we conducted a wild‐GWAS to identify genetic variants associated with this colour variation. We discovered 65 genes containing variants that significantly correlate with the chroma of the elytra, including five genes with known roles in cuticle pigmentation processes (Figure 3). Among these five genes, *yellow‐e* and *pdm3* in the tyrosine‐mediated tanning pathway are most likely to contribute to the colour variation in the *N. ingens* complex, as the multilayer reflectors are primarily composed of alternating layers with different degree of melanin deposition (Hamza and Zahran 2023; Onelli et al. 2017; Parker et al. 1998). Yellow protein (dopachrome conversion enzyme) from the *yellow* gene family advances melanin biosynthesis, a vital biochemical pathway altering the insect cuticle profile by converting tyrosine into pigments (e.g., melanin and dopamine‐melanin) (Arakane et al. 2016; Noh et al. 2016). This gene has been identified as a key mediator of the melanin biosynthesis pathway across multiple insect species (Gompel et al. 2005; Li and Christensen 2011; Noh et al. 2015; Zhang, Martin, et al. 2017). On the other hand, the transcription factor *pdm3* is known for its role in regulating neural development, but it has also been identified as a repressor of abdominal pigmentation in *Drosophila melanogaster* , potentially through co‐expression with other melanin biosynthesis transcription factors such as *Abdominal‐B* and *doublesex* (David et al. 2026; Hughes et al. 2023; Kim and Cho 2020; Rogers et al. 2014; Yassin et al. 2016). However, the function of *pdm3* orthologs in beetle (Coleoptera) species remains unexplored, highlighting the need for further studies that investigate its role in beetle cuticle pigmentation. In contrast, *chico* and *PK1‐like* genes are indirectly involved in cuticle pigmentation through the Insulin/IGF and pyrokinin signalling pathways, respectively (Matsumoto et al. 1990; Shakhmantsir et al. 2014). Although their involvement is indirect, signals of positive selection from the selective sweeps have been previously identified in these genes in the *N. ingens* complex, underscoring the importance of further investigating their roles in the pigmentation of beetle elytra (Weng et al. 2024). Finally, *aristaless*, a transcription factor known to influence appendage formation and scale colour in butterflies, has been identified as a candidate gene (Bayala et al. 2023). However, *aristaless* is involved in ommochrome pigment production through the ommochrome pathway. Since melanin is the dominant pigment in insect cuticles, we consider *aristaless* the least likely candidate among the five. In summary, we identified five genes linked to beetle elytra colour, with *yellow‐e* and *pdm3* most likely influencing melanin‐based pigmentation. *Chico* and *PK1*‐like show indirect roles and signs of selection, while *aristaless*, involved in a different pigment pathway, is the least likely candidate.

### 4.2 The Evolutionary and Ecological Role of Metallic Colour in the *N. ingens* Complex

The ecological function of metallic colour has been considered an important characteristic in biotic interactions, both within and between species (Clusella‐Trullas and Nielsen 2020). In beetles, sexual selection, mimicry, camouflage, or aposematism are widely discussed functions for metallic colour (Kjernsmo et al. 2022; Lee et al. 2018; Vulinec 1997; Xu et al. 2010). In the *N. ingens* complex, however, the relationship between metallic colour variation and biotic interactions is less straightforward. While further research is warranted, abiotic factors and local adaptation may provide a more plausible explanation for the observed colour variation. No assortative mating was observed in the no‐choice mating trials between lineages of *N. ingens* complex, even when individuals differed in coloration, suggesting that colour variation may not play a major role in mate recognition (Weng, Kavanaugh, and Schoville 2021; Weng, Francoeur, et al. 2021). As nocturnal predators and scavengers occupying relatively high trophic positions within nival‐zone communities, these beetles likely experience relatively low predation pressure, although the metallic colour might serve as a form of camouflage mimicking water in wet microenvironments. In general, it remains unclear whether biotic interactions explain the diversity of colour patterns in beetles (Ospina‐Rozo et al. 2024; Schultz and Hadley 1987). Other studies have shown that metallic colour may instead result from adaptation to abiotic environmental factors (Davis et al. 2008; Keinath et al. 2020). In particular, pigmentation from the melanization pathway in the epidermal and serosal cuticle is thought to be associated with desiccation resistance (Bai et al. 2022; Farnesi et al. 2017; Wang et al. 2021). In the subgenus *Catonebria*, which includes the *N. ingens* complex, metallic colour variation has been gained and lost many times with a general trend that metallic species occur in cooler and wetter regions compared to related species with darker, less or non‐metallic cuticle. For example, among species of the *Nebria gebleri* species complex (Kavanaugh et al. 2021), those from more northern and coastal regions (northern Cascade Range and northern Rocky Mountains; namely, *N. gebleri* Dejean and *N. cascadensis* Kavanaugh) are more vividly metallic than those from the Sierra Nevada and other southern ranges in California (i.e., *N. albimontis* Kavanaugh, *N. rathvoni* LeConte, and *N. siskiyouensis* Kavanaugh). The same general pattern is seen among members of the *meanyi* species complex as well as in other closely related *Nebria* subgenera. To investigate this phenomenon, we explored the possibility that metallic variation might be associated with water availability in *N. ingens* habitats, with the expectation that the black individuals could have higher fitness in drier and warmer habitats. Evidence from other beetle species has shown a relationship between the *yellow* gene, cuticle melanization and water retention ability (Noh et al. 2015). This leads to the hypothesis that the loss of metallic colour, as a byproduct of melanin accumulation, is the result of adaptation to arid environments. Our results show significant correlations between habitat SWE and chroma (beetle coloration), indicating that less melanized beetles are more commonly found in habitats with lower snow covers (Figure 4A). Moreover, individuals inhabiting these drier environments tend to exhibit better water retention ability. However, the correlation between chroma and water loss rate is moderate and not statistically significant (Figure 4B), suggesting that additional factors could be contributing to variation in water retention ability.

### 4.3 Study Limitations and Future Directions

While our wild‐GWAS approach successfully identified genetic variants associated with beetle elytra colour variation, several limitations may have influenced the strength of the association signals. Despite efforts to minimize noise, challenges in accurately quantifying colour, such as measurement errors due to varying specimen conditions, could have introduced variability into the data. Additionally, nearly half of the candidate genes predicted from the reference genome lack functional annotation, which means that important genes may have been overlooked in our analysis. Nonetheless, we identified five promising candidate genes that warrant further functional investigation to better understand their roles in producing the metallic coloration in beetles.

## Author Contributions

Y.‐M.W., D.H.K. and S.D.S. developed the original idea; Y.‐M.W. and S.D.S. designed the experiments. The quantification of colour variation was conducted by Y.‐M.W., B.R.‐P. and J.S. and other analyses were performed by Y.‐M.W. Y.‐M.W. and S.D.S. wrote the first draft of the manuscript, and D.H.K., B.R.‐P., J.S. and M.A.K. provided comments to improve the manuscript.

## Funding

This work was supported by Valentine Eastern Sierra Reserve. Ministry of Education of the Republic of China (Taiwan).

## Ethics Statement

The present study complies with all applicable institutional and national guidelines for the sampling of natural populations. Institutional ethics approval was not required for this study, as the *Nebria ingens* complex consists of insect species that are not subject to animal ethics committee regulations.

## Conflicts of Interest

The authors declare no conflicts of interest.

## Supporting information


**Figure S1:** The calculated reflection spectra from different types of multilayer reflectors in the epicuticle (from left to right: three electronic dense layers, two electronic dense layers and one electronic dense layer). The beetle on the left shows the visual colour of the elytra. Three types of multilayer reflectors (images on the top‐right) with their calculated reflection spectrum (black lines) are shown with the observed spectrum (green line).
**Figure S2:** Scatter plot of BH adjusted *p*‐values of outlier SNPs from ridge (x‐axis) and lasso (y‐axis) regressions in the LFMM analysis.

**Table S1:** Beetle sample metadata including sample ID, taxonomy and collecting information.
**Table S2:** Relatedness of beetle samples with and without SNP pruning. The pruning was performed using snpgdsLDpruning function with default setting.
**Table S3:** Measured reflectanc specturm using hyperspectral camera (Specim IQ).
**Table S4:** Converted CIELAB colour spaces from the measured reflectance spectra.
**Table S5:** Thickness of visible layers in the epicuticle of three N. riversi complex individuals under TEM.
**Table S6:** Calculated reflectance spectrum from the layer thicknesses measured under TEM images (data in Table S3).
**Table S7:** Outlier SNP loci from the LFMM and partial RDA analyses.
**Table S8:** List of functionally annotated genes that intersect colour_correlated candidate variants. Genes related to cuticle pigmentation are bolded.

## Data Availability

Image data, research note, morphological and physiological measurements and genotype data are available on https://doi.org/10.6084/m9.figshare.33099305.
