# Supplementary material for: The Genomic Landscape of Metallic Colour Variation in Ground Beetles
Source: Mol Ecol. 2026 Oct 16;35(20):e70591. doi: 10.1111/mec.70591 (PMC13675647; doi:10.1111/mec.70591)
Supplement: Supplementary file 1 [file MEC-35-e70591-s002.docx]

Supplemental Information for

**The genomic landscape of metallic color variation in ground beetles**

Yi-Ming Weng^1,4^*, David H. Kavanaugh^2^, Bryan Rubio-Perez^3^, Jad Salman^3^, Mikhail A. Kats^3^, Sean D. Schoville^1^

1. University of Wisconsin-Madison, Department of Entomology, Madison, WI, USA

2. California Academy of Sciences, Department of Entomology, San Francisco, CA, USA

3. University of Wisconsin-Madison, Department of Electrical and Computer Engineering, Madison, WI, USA

4. University of Wisconsin-Madison, Department of Physics, Madison, WI, USA

5. University of Wisconsin-Madison, Department of Material Science and Engineering, Madison, WI, USA.

6. Okinawa Institute of Science & Technology Graduate University, 1919-1 Tancha, Onna-son, 904-0495 Okinawa, Japan

* Corresponding author: Yi-Ming Weng ([weng76111@gmail.com](mailto:weng76111@gmail.com.edu))

**Supplemental methods**

*Variants filtering and indel recoding and genotype data conversion*

Single-nucleotide polymorphism (SNP) and insertion/deletion (indel) variants were filtered using GATK v4.1.9.0 with the VariantFiltration function (Van der Auwera & O’Connor, 2020). SNPs were hard-filtered based on variant-level quality metrics, and variants failing any of the specified criteria were flagged as low quality. The following thresholds were applied: quality by depth (QD) < 5.0, variant quality (QUAL) < 30.0, strand odds ratio (SOR) > 1.1, Fishers strand bias (FS) > 7.0, mapping quality (MQ) < 59.8, mapping-quality rank-sum statistic (MQRankSum) < −0.1, or read-position rank-sum statistic (ReadPosRankSum) < −0.1. Variants passing all filters were retained for subsequent analyses. Indels were filtered separately using GATK VariantFiltration. Indels were flagged when QD < 7.0, QUAL < 30.0, FS > 7.0, or ReadPosRankSum < −0.1. The filtered SNP and indel datasets were subsequently combined for downstream genotype analyses.

Following variant filtering, indel alleles were recoded to a standardized biallelic representation using A/G as the reference/alternate allele coding. The resulting SNP and indel genotype dataset was converted from VCF format to PLINK binary-compatible text format, consisting of .map and .ped files, using VCFtools v0.1.16 (Danecek et al., 2011). The resulting files (snp_indel_gwas.map and snp_indel_gwas.ped) were used as the input genotype dataset for downstream population genetic and genotype-based analyses.

*Genotypic data preparation for LFMM*

We used the single nucleotide polymorphism (SNP) data matrix generated from our previous study (Weng et al., 2024) as the genetic marker to perform GWAS using latent factor mixed models (LFMM). The SNP matrix containing 526,717 SNP+indel variants was originally called from 374 beetle genomes, with series of filtering preprocessed in the previous study and stored in the variant call format (vcf) file, including the removal of loci with minor allele frequency less than 0.05. In this study, we first subset the variant matrix to 355 beetle individuals with color variation data using SelectVariants function in GATK (Van der Auwera & O’Connor, 2020). The subset SNP matrix was subsequently converted to ped format using vcftools v0.1.16 for LFMM (Danecek et al., 2011).

*Quantification of Color Variation*

Metallic color variation of adult beetles varies as a reflection spectrum on the elytra. We measured reflectance using a hyperspectral camera (Specim IQ) to record every pixel in a dorsal image of each ethanol preserved specimen (n=368), comprised of a 512 × 512 pixel array. The spectral resolution of the image is 7 nm and the spectrum ranges from 400 to 1,000 nm, with 204 spectral wavelength points in this range. The images were then used to extract the refection spectrum by manually selecting the brightest region of the elytra (because the elytral surface is convex and depends on the location of the input light). Selected areas consisted of 200 pixels on average (8-600 pixels, median=190 pixels), and the mean reflectance was calculated from the selected pixels for each of the 204 spectral wavelength points. Finally, we used the mean reflection spectrum to calculate the CIE LAB color space for each elytron using CIE standard illuminant D65 (Robertson, 1977), in which we used axis a* (the axis of green-red) to represent the color of the elytral sample. We chose this color axis because it encompasses the most variation in elytral color.

*Cross-section TEM of elytra and calculated reflectance spectra*

From the cross-section TEM images of the green, dark green, and black elytra, we found multilayer reflectors embedded in the inner epicuticle (cuticulin). This structure was found to vary within the green elytra, where the reflector with 2 electron dense layers is most commonly seen comparing to the reflectors with 1 and 3 electron dense layers. We thus used the reflector with 2 electron dense layers to represent the green elytra. For modeling the layers, we repeatedly measured each layers including the top thick electron dense area at 26-30 points (green: 30 points, dark green: 27 points, and black: 26 points) along the elytral surface using ImageJ (Abràmoff et al., 2004). The lengths of the layers were subsequently used to calculate the expected reflection spectrum using reflectance calculator provided by Corporation Filmetrics®.

**References**

Abràmoff, M. D., Magalhães, P. J., & Ram, S. J. (2004). Image processing with ImageJ. *Biophotonics International*, *11*(7), 36–42.

Danecek, P., Auton, A., Abecasis, G., Albers, C. A., Banks, E., DePristo, M. A., Handsaker, R. E., Lunter, G., Marth, G. T., Sherry, S. T., McVean, G., & Durbin, R. (2011). The variant call format and VCFtools. *Bioinformatics*, *27*(15), 2156–2158. https://doi.org/10.1093/bioinformatics/btr330

Robertson, A. R. (1977). The CIE 1976 Color-Difference Formulae. *Color Research & Application*, *2*(1), 7–11. https://doi.org/10.1002/j.1520-6378.1977.tb00104.x

Van der Auwera, G. A., & O’Connor, B. D. (2020). *Genomics in the cloud: Using Docker, GATK, and WDL in Terra*. O’Reilly Media.

Weng, Y.-M., Kavanaugh, D. H., & Schoville, S. D. (2024). Evidence for admixture and rapid evolution during glacial climate change in an alpine specialist. *Molecular Biology and Evolution*, *41*(7), msae130. https://doi.org/10.1093/molbev/msae130

**Supplemental Figures**

**Figure S1.** The calculated reflection spectra from different types of multilayer reflectors in the epicuticle (from left to right: three electronic dense layers, two electronic dense layers, and one electronic dense layer). The beetle on the left shows the visual color of the elytra. Three types of multilayer reflectors (images on the top-right) with their calculated reflection spectrum (black lines) are shown with the observed spectrum (green line).

**Figure S2.** Scatter plot of BH adjusted p-values of outlier SNPs from ridge (x-axis) and lasso (y-axis) regressions in the LFMM analysis.
